# Giant laryngopharyngeal lipoma

**DOI:** 10.1016/j.bjorl.2024.101407

**Published:** 2024-02-21

**Authors:** Bo Li, Xiaoming Fan, Delong Liu, Yang Song, Cuiping She

**Affiliations:** a Dalian Municipal Central Hospital, Department of Otorhinolaryngology Head and Neck Surgery, Dalian, China; b Dalian Medical University, Dalian, China; c Dalian Municipal Central Hospital, Department of Pathology, Dalian, China; d Dalian Municipal Central Hospital, Radiology Department, Dalian, China

## Introduction

A lipoma is a benign tumor derived from mesenchymal cells that is most commonly found within the subcutaneous tissues of the limbs and trunk. However, rare cases of lipomas situated within the hypopharynx have been reported that comprise approximately 0.6% of all benign hypopharyngeal tumors. In his 1995 review of laryngopharyngeal lipoma cases, Wenig summarized three new cases along with approximately 80 previously reported cases found within the literature.^1^ Since then, the total number of reported laryngeal and hypopharyngeal lipoma cases has increased to almost 100 cases to date.^2^ This concise review, which focuses on clinical information related to one new hypopharyngeal case treated at our hospital and previously reported laryngopharyngeal lipoma cases, provides a comprehensive overview of the diagnosis and treatment process for individuals with laryngopharyngeal lipomas.

## Case report

**Fig. 1:**
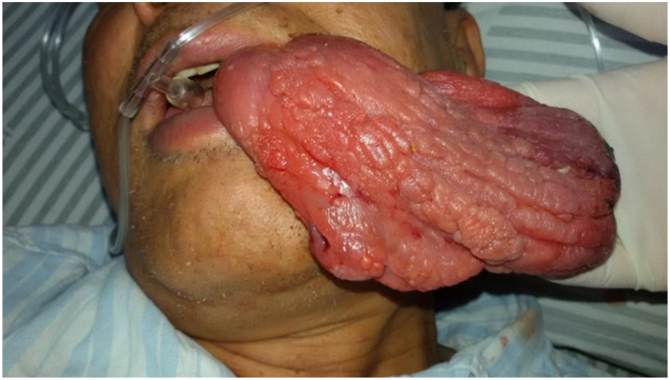
The size of the extra-oral mass was about 15 × 6 × 4 cm.

**Fig. 2:**
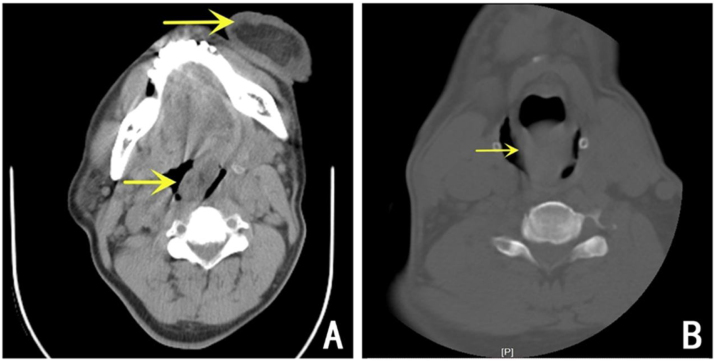
(A) Preoperative CT showed that the upper respiratory tract in the oropharynx and throat was occupied by soft tissue density and extended into the mouth. (B) Preoperative CT showed a huge, large mass in the laryngopharynx with visible dilatation of the esophageal entrance.

**Fig. 3:**
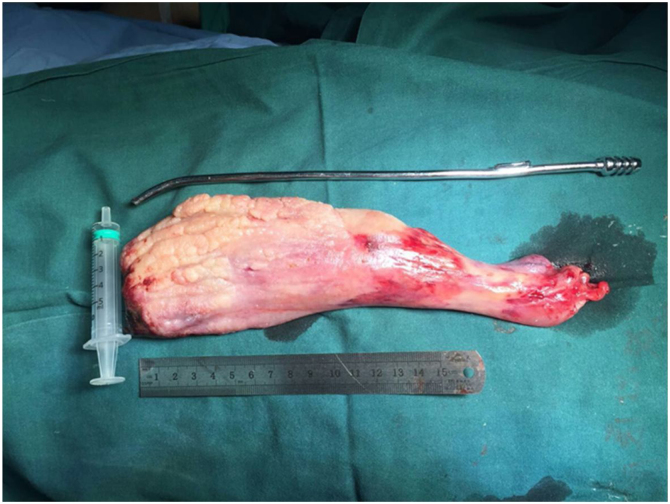
The size of the resected mass during the operation was approximately 20 × 6 × 4 cm.

**Fig. 4:**
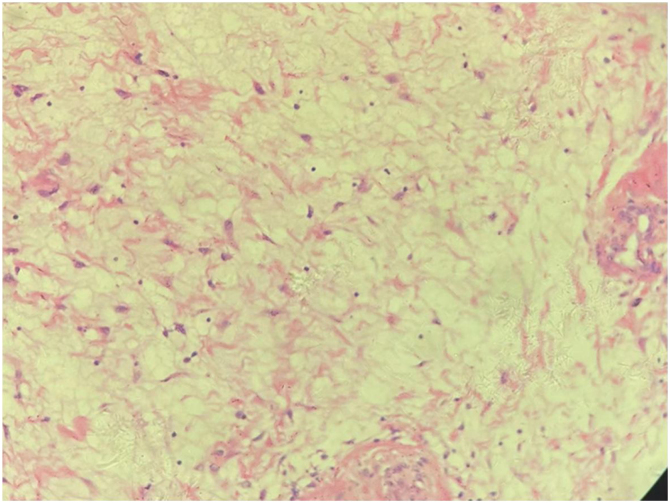
Postoperative pathological findings revealed hyperplasia of adipose tissue (hypopharynx), with a partial envelope, consistent with lipoma. The surface surrounding the tumor was partially covered with squamous epithelium, accompanied by inflammation, and inflammatory necrotic tissue.

**Fig. 5:**
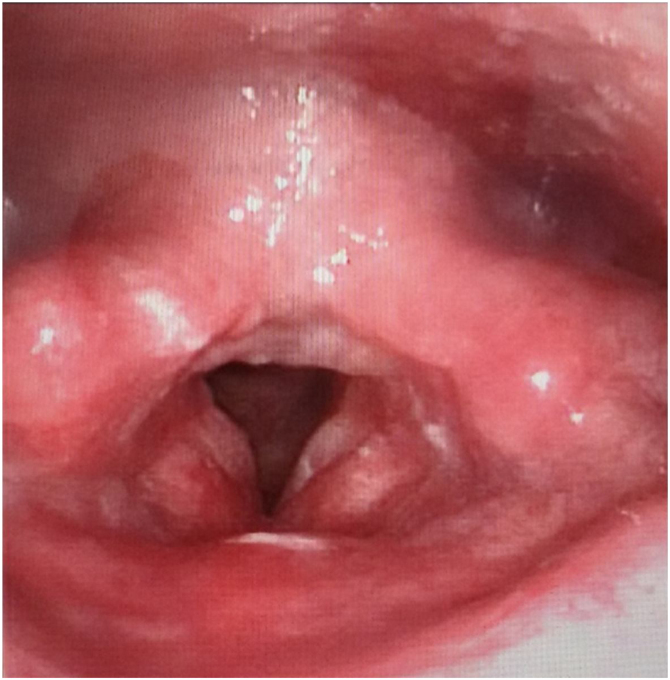
After 8-years of follow-up, laryngoscopy showed no recurrence of mass.

A 58-year-old male presented to the emergency room with dyspnea and a red mass protruding from his mouth. Upon examination, the patient was conscious with stridor, and his blood oxygen saturation was at 90%. The extraoral mass was about 15 × 6 × 4 cm in size, soft, and showed no tenderness (Fig. 1). Computed Tomography (CT) scanning of the mass revealed that the oropharynx and upper pharyngeal respiratory tract harbored dense soft tissues extending into the oral cavity, while the normal structure of the left pyriform fossa was no longer visible (Fig. 2). Given the patient's respiratory difficulty and the urgency of the situation, a tracheotomy under local anesthesia was initially performed. Subsequently, a gastroscopy under general anesthesia was conducted to locate the base of the tumor. The tumor originated near the entrance of the esophagus from the posterior cricoid mucosa on the left side, with a broad base. The middle and lower sections of the esophageal mucosa appeared smooth with evident dilation of the lumen. Using a multidirectional laryngeal retractor and a 30 ° endoscope for visualization, the tumor was grasped with laryngeal forceps and progressively excised from its base using a #5874 low-temperature plasma knife (Arthrocare Corporation, set at cutting level 7 and coagulation level 5), ensuring complete removal of the mass. The intraoperative bleeding was minimal, and the surgical field was clear. The dimensions and mass of the resected tumor were about 20 × 6 × 4 cm and 0.29 kg, respectively (Fig. 3). Postoperatively the patient was fed nasally for 14 days, during which the patient repeatedly tested negative for active bleeding recurrence or esophageal perforation after undergoing multiple electronic laryngoscopy examinations. Thereafter, oral feeding was resumed. Pathological findings were consistent with lipoma (Fig. 4). No lipoma recurrence was observed during the 8-year postoperative follow-up period (Fig. 5).

## Discussion

Lipomas of the hypopharynx and larynx are very rare. A comprehensive review of the global lipoma-related literature indicated that laryngopharyngeal lipomas predominantly range in size from a few millimeters to 6 cm.^1^ Remarkably, the size of the 20-cm-long lipoma detected in the case, as documented in this report, surpasses that of the largest previously reported 18-cm-long^3^ laryngopharyngeal lipoma and thus is the largest laryngopharyngeal lipoma reported to date. Most patients experience mild but persistent long-term symptoms. Significantly, laryngopharyngeal tumor growth can lead to severe complications, including sudden airway obstruction that potentially results in death by asphyxiation, as reported in this study's case, where the patient's respiratory distress symptoms required urgent medical attention. Therefore, early detection of laryngopharyngeal tumors calls for prompt surgical intervention.

Owing to the rarity of lipomas, they are often misdiagnosed as other benign lesions, such as mucoceles or laryngeal bulges, especially since these conditions share similar laryngoscopic views and cause analogous symptoms. Imaging techniques like CT scans and MRI can easily distinguish lipomas from these conditions. CT scans are preferred for their faster acquisition and easier accessibility. Lipomas typically appear on CT as non-enhancing, homogeneous, well-demarcated lesions with low radiodensity, less than that of water. The diagnostic accuracy of CT is between 75% and 90%. In the case reported in this article, due to the urgency of the situation, MRI was not performed; instead, a more expedient CT scan was used for preoperative assessment.

Radical excision is the treatment of choice for laryngopharyngeal lipomas, given that most lipomas are encapsulated within envelopes that thereby facilitate complete resection of the tumors. Meanwhile, in recent years surgical lipoma resection has been achieved using low-temperature plasma technology,^4^ which utilizes a plasma knife to maintain hemostasis during the procedure to maintain a clear operative field, while the low temperature maintained during the procedure minimizes damage to surrounding tissues. Although the use of low-temperature plasma technology for tumor removal is relatively safe, careful operation is imperative due to the large size, abundant blood supply, and broad base of the tumor, as well as the difficulty in exposing the pedicle. This careful approach is necessary to avoid complications such as excessive bleeding, mucosal damage, and potential esophageal perforation.

## Conclusion

In conclusion, laryngopharyngeal lipomas are rare in clinical practice. Herein we report the largest laryngopharyngeal lipoma reported to date. Literature review indicates that while most laryngeal lipoma patients experience mild symptoms, there is a risk of rapid airway obstruction. Tracheotomy is imperative in cases of respiratory distress. The hemostatic capability of plasma knives offers a distinct advantage in tumor treatment. It is advisable to opt for plasma surgery when the laryngopharyngeal surgical field is well exposed, and the tumor base is clearly visible. Furthermore, postoperative pathology should be carefully evaluated to differentiate lipomas from highly differentiated liposarcomas, while long-term post-surgical follow-up monitoring of tumor recurrence should be conducted to improve patient treatment outcomes.^5^

## Statements and declarations

This research did not receive any specific grant from funding agencies in the public, commercial, or not-for-profit sectors.

## Conflicts of interest

The authors declare no conflicts of interest.
